# Study of Operative Outcomes of Hernioplasty Using Mosquito Net Mesh at a Tertiary Hospital

**DOI:** 10.7759/cureus.28525

**Published:** 2022-08-29

**Authors:** Niteen N Chate, Nagargoje S Motiram, Bhushan M Jogdand, Mahesh D Birajdar

**Affiliations:** 1 Department of General Surgery, Swami Ramanand Teerth Rural (SRTR) Govt. Medical College, Ambajogai, IND

**Keywords:** polypropylene mesh, synthetic mesh, mosquito net mesh, hernioplasty, inguinal hernia

## Abstract

Background

Synthetic mesh is the most efficient way to repair groin hernias. However, many patients in low and middle-income countries cannot afford the above-mentioned mesh. This study's goal was to describe the mosquito net mesh's (MNM) safety, viability, complications, and recurrence rate in hernioplasty done in rural populations.

Material and methods

This study was a single-centre, prospective, descriptive study, conducted in patients >18 years of age, of either gender, with unilateral or bilateral primary inguinal hernias (reducible/irreducible/bilateral hernia), admitted for elective or emergency open inguinal hernia mesh repair. The surgery was performed using reasonably priced (affordable to people of low socioeconomic status) polyethylene mosquito net mesh, which was cut into 8x16 cm rectangles and wrapped in two sealed plastic bags. They were sterilised using the ethylene oxide sterilisation* *(EtO) method. The type of hernia (unilateral or bilateral), post-operative pain, and complications were noted.

Results

Among 400 study participants, the incidence of inguinal hernia was highest in the 60-79 year age group (51.5%). Direct inguinal hernia (67.5 %) was higher compared to indirect inguinal hernia (32.5 %). While a majority of the participants had a hernia on the right side (50%), 164 patients (41%) had a hernia on the left side, and 36 patients (9%) had a bilateral inguinal hernia. The average operative time for unilateral inguinal hernia was 73.96 minutes and for bilateral inguinal hernia was 106.66 minutes. Out of 400 patients, 355 patients (88.75%) had no complications. Among the postoperative patients, 14 patients (3.5%) experienced surgical site infection; 9 patients (2.25%) experienced headache; 5 patients (1.25%) experienced hematoma; 12 patients (3%) experienced urinary retention; and 5 patients (1.25%) experienced testicular pain. The average hospital stay of patients was 4.25 days. Patients returned to their daily activities and employment in an average of 7.29 days. The market cost of a single standard polypropylene mesh was Rs.1,660/-. For making a single mosquito net mesh of the same size, we had an expenditure of Rs. 11.83/- including the cost of sterilization. In this study, 382 patients (95.5%) gave us good feedback, 11 patients (2.75) gave satisfactory feedback, and 7 patients (1.75%) gave excellent feedback.

Conclusion

In environments with limited resources, using mosquito net mesh for hernioplasty is reasonable, acceptable, doable, and economical.

## Introduction

A hernia is the protrusion or prolapse of a viscus or a portion of a viscus through an abnormal opening in the walls of its enclosing cavity. Hernia repair is the most common surgical procedure carried out worldwide. The Lichtenstein repair is the most commonly practised all-open inguinal hernia repair method [1]. A tension-free repair, reduced pain, a decreased recurrence rate, and quick recovery are all benefits of the mesh repair procedure. In poor and underdeveloped nations, the majority of patients cannot afford meshes or cannot access them [2].

Although abdominal hernia is a prevalent and expensive public health issue, there is a cost-effective remedy. The use of polypropylene mesh as a prosthesis is currently the gold standard for hernia repair in developed nations [3]. Because commercial prosthetic mesh is scarce and expensive, the traditional Bassini operation is still carried out in the majority of locations in poor countries.

The Government of India approved the Ayushman Bharat-Pradhan Mantri Jan ArogyaYojana (AB-PMJAY) in March 2018 [4]. Successive Indian governments have stated a commitment to achieving universal health coverage (UHC). Despite this, UHC remains an elusive aim and the Indian health system continues to be characterised by substantial shortcomings relating to the workforce, infrastructure, and the quality and availability of services. Public expenditure on health care in India remains at levels amongst the lowest in the world [4]. While many have already questioned the likelihood of successful implementation of the AB-PMJAY, the vast ambition of the programme presents an opportunity to pursue the systemic reform that India requires to meet its UHC aims [4]. This will require an injection of resources into a chronically underfunded health system, but this must be accompanied by a focus on the interrelated issues of governance, quality control, and stewardship if the scheme is to sustainably accelerate India towards UHC.

Synthetic mesh is the most efficient way to repair groin hernias. However, many patients in low and middle-income countries cannot afford this kind of expensive mesh. Although not thoroughly researched, sterilised mosquito mesh has been utilised as a less expensive option. It must be acknowledged that Tongaonkar & Sanders were responsible for spreading the innovative idea of utilising mosquito net mesh in open inguinal hernia surgery in India [5]. The goal of this study was to evaluate the cost-benefit of regular polypropylene mesh repair with mosquito net mesh (MNM) in rural areas (among patients of lower socioeconomic strata) while elaborating on safety, practicality, problems, and recurrence rates.

## Materials and methods

The current study was a prospective, single-centre, descriptive investigation carried out at the Department of General Surgery at the SRTR Medical College in Ambajogai, India. The study was conducted from July 2018 to June 2019. The institutional ethical committee approved the study with letter number "IEC/122/2018", dated February 12, 2018.

All patients over the age of 18 years of any gender, having unilateral or bilateral primary inguinal hernias (reducible, irreducible, or bilateral hernias), admitted for elective or emergency open inguinal hernia mesh repair, and willing to participate in the study were included in the study. However, patients under the age of 18 years with a recurrent or strangulated hernia resulting in bowel obstruction and patients not consenting to the procedure were excluded.

Informed consent was acquired after the study was fully explained. Information on demographics, history, and clinical findings was recorded. For surgery, easily available, polyethylene mosquito net mesh that was not insecticide-impregnated was employed. The mesh was cut into 8x16 cm rectangles and wrapped in two sealed plastic bags. They were sterilised using the ethylene oxide (EtO) sterilisation process. Sterilisation was then confirmed using a quality control colour indicator. After three weeks, the meshes were no longer considered sterile and could not be used. The sterilisation did not affect the mesh's macroscopic structure on gross visualisation. Low-density polyethylene woven into several filaments was used to make the implants.

The typical Lichtenstein procedure for hernia repair was carried out under spinal anaesthesia. A prophylactic single intravenous antibiotic dose was administered before the incision. The same pair of general surgeons worked together to complete every operation. Standard postoperative care was given and a one-month follow-up was maintained. Feedback was also obtained from the patients and marked as excellent, good, and satisfactory [4].

IBM USA's Statistical Package for Social Sciences (SPSS) version 25.0 and Microsoft Excel were used to gather and compile the data. The type of hernia (unilateral or bilateral), post-operative pain, and complications were noted. Descriptive statistics were used in the statistical analysis, and all the data was compiled as numbers (percentages).

## Results

Among 400 study participants, the incidence of inguinal hernia was the highest in the older age group of 60-79 years (51.5%), followed by the age groups of 40-59 years (26.75%), 20-39 years (18.5%) and 80 years (3.25%), respectively. The most common comorbidity was hypertension (19.5%), followed by chronic obstructive pulmonary disease (COPD) (10%). The number of patients with no comorbidities was 282 (70.5%) (Table 1).

**Table 1:** The age groups and co-morbidities of participants COPD: chronic obstructive pulmonary disease; HTN: hypertension

Variables	Frequency (n=400)	Percentage (%)
Age group (in years)		
20-39	74	18.5
40-59	107	26.75
60-79	206	51.5
80-89	13	3.25
Comorbidities		
COPD	40	10
HTN	78	19.5
No comorbidity	282	70.5

In the present study, the incidence of direct inguinal hernia (67.5 %) was high compared to indirect inguinal hernia (32.5 %). While the majority had a hernia on the right side (50%), 164 patients (41%) had a hernia on the left side, and 36 patients (9%) had a bilateral inguinal hernia (Table 2).

**Table 2:** Type and location of the hernia

Variables	Frequency (n=400)	Percentage
Type of hernia		
Direct hernia	270	67.5
Indirect hernia	130	32.5
Location of hernia		
Right side	200	50
Left side	164	41
Bilateral	36	9

The average operative time for unilateral inguinal hernia was 73.96 minutes and for bilateral inguinal hernia, the average operative time was 106.66 minutes (Table 3).

**Table 3:** Average operative time

Type of hernia	Operative time (in minutes)
Unilateral hernia	73.96
Bilateral hernia	106.66

Postoperative pain was recorded at 24 hours, wherein 365 patients (91.25%) had mild pain, 31 patients (7.75%) had moderate pain, and 4 patients (1%) had severe pain (Table 4).

**Table 4:** Postoperative pain

Postoperative pain	Frequency(n=400)	Percentage (%)
Mild pain	365	91.25%
Moderate pain	31	7.75%
Severe pain	4	1%

Postoperative complications were found in 45 (11.25%) of the patients. Out of 400 patients, 364 patients (91%) had no complications, while 14 patients (3.5%) had surgical site infection, 5 patients (1.25%) had hematoma, 12 patients (3%) had urinary retention, and 5 patients (1.25%) had testicular pain (Table 5).

**Table 5:** Postoperative complications

Postoperative complications	Frequency (n=400)	Percentage
Surgical site infection	14	3.5
Urinary retention	12	3
Hematoma	5	1.25
Testicular pain	5	1.25
No complications	364	91

The average hospital stay of patients was 4.25 days. Patients returned to their daily activities on an average of 7.29 days. The market cost of a single standard polypropylene mesh was Rs.1660/-. To make a single mosquito net mesh of the same size, we had an expenditure of Rs.11.83/-, including the cost of sterilisation (Table 6).

**Table 6:** Other characteristics

Other characteristics	
Average hospital stay	4.25 days
Return to activity	7.29 days
Mesh type	Cost (in rupees)
Polypropylene	Rs.1600
Mosquito net mesh	Rs.11.83

In this study, 382 patients (95.5%) gave us good feedback, 11 patients (2.75) gave us satisfactory feedback, and 7 patients (1.75%) gave us excellent feedback (Table 7).

**Table 7:** Patient feedback

Patient feedback	Frequency(n=400)	Percentage (%)
Excellent	7	1.75
Good	382	95.5
Satisfactory	11	2.75

## Discussion

In 1949, polyethylene was introduced as the first synthetic surgical thread and it has been in use ever since. It is used for several types of sutures. A sterilised polyethylene fishing line from the local market is used by surgeons in some low-middle income countries (LMIC) where the commercial surgical thread is not readily available [6,7].

In developed nations, several mesh types are frequently used to treat hernias. Research is being done extensively to determine the best mesh textures and materials. The most widely used material is polypropylene, followed by polyester. Polypropylene causes substantial fibrosis but a less severe chronic inflammatory response than other materials. It is inert and maintains its tensile strength over time [8]. The drawbacks have been noted as shrinking and stiffness, along with a foreign body sensation [9]. Recent clinical and experimental results indicate that these drawbacks may be solved by lightweight meshes with more pores and less material, possibly in combination with polyglactin fibres [9,10].

In his study, Jindal emphasised the need for training in healthcare settings. The training should not only focus on patient diagnosis, treatment, and management, but also one should feel the joy behind it. The word "Seva" is of Sanskrit origin, which means the performance of duties and serving mankind without the greed for reward or gifts. Similarly, the burnout of surgeons in hernia repair can be taken care of if the repair is done with full dedication and commitment [11]. In another study conducted by Vora et al., they also concluded that by using a mixed methodology, the deficiency of trust from the patients can be mitigated by good communication between doctor and patient, particularly that all patients should be managed equally irrespective of their paying capacity [12].

Sorensen and Rosenberg, who conducted a systematic assessment of the use of sterilised mosquito nets for hernioplasty, found their use was extremely cost-effective and ought to be encouraged in environments with limited resources. However, they issued a warning that it was unclear what the outcome would be in the long run [13].

The longest inguinal hernioplasty follow-up to date is presented by Tongaonkar et al. in their paper, which details results for up to 10 years [14]. They treated 651 patients for 713 inguinal hernia repairs, using inexpensive polyethylene mesh during the research period. The majority of patients had access to follow-up questionnaires at six to 18 months. However, 32 (4.9%) of those patients were not available for follow-up. During postoperative monitoring, six patients had superficial surgical site infections (0.9%), one had seroma (0.1%), two patients complained of persistent pain (0.3%), and two had hematomas (0.3%). All of these conditions were successfully treated with the appropriate antibiotics. In this research, there were no instances of mesh rejection or recurrence of a hernia. There is little danger of infection from the mosquito-net mesh itself since it is comprised of monofilament fibres, but they are less likely to harbour bacteria than braided or multifilament materials [15].

Four trials utilising inexpensive mesh were examined by Yang et al. in their study [16]. Three investigations examined the postoperative results for repairs made using commercial surgical mesh and those made with sterile mosquito nets. They came to the conclusion that there were no appreciable changes in the results between the two types of repairs.

By analysing the crucial (first) stage in the pathogenesis of mesh infections, Sanders et al. evaluated the infection risk of polyethylene (PE) mosquito net mesh compared with commercial hernia prosthetics in their study. They discovered there was no significant difference in the mean number of adherent bacteria in the PE mosquito net compared with the monofilament polypropylene-based meshes [17,18]. A systematic review and meta-analysis were undertaken by Patterson et al., who came to the conclusion that there was no discernible difference between the commercial mesh group and the mosquito net mesh group [19].

In a study by Edwin M. T. Yenli et al., out of 184 patients who underwent surgery using a mosquito net mesh, only one patient had a strangulated hernia and didn't need to have their bowels removed. In 6.7% of our patients, a scrotal hematoma developed after surgery [20]. A purulent discharge from the site was seen in about 2.9% of the trial participants, but this was successfully treated with antibiotics.

M. G. Clarke et al. investigated 95 cases where mosquito net meshes were used [21]. There were seven wound problems noted at the six-week follow-up. These included two small wound infections that were effectively treated with antibiotics and five wound hematomas, one of which required surgical evacuation. According to the reports, there were no incidences of recurrence or mesh rejection at six months in his study. In our 400-patient trial employing mosquito net mesh, there were no recurrences or instances of mesh rejection throughout the study period.

Our results are in line with earlier observational studies that found similar outcomes in terms of postoperative complications and hernia recurrence between patients undergoing groin hernia repair using a low-cost mesh and those undergoing the procedure using a commercial mesh. Our results are also in line with a large-scale clinical experience with low-cost mesh [15,22].

In their study, Tongaonkar et al. discovered that 7.5 x 15 cm of mosquito-net cloth costs only 45 paise (as opposed to the synthetic commercial mesh for hernia repair available on the market which costs Rs.1,666/-); 15 x 15 cm costs 90 paise (as opposed to Rs.3724/-); and 30 x 30 cm costs just Rs.3.60/- (as opposed to Rs.9,430/-). It is highly advised for LMIC nations like India to utilise this material for hernia repair [14]. The conclusions/inferences from different researchers are tabulated below (Table 8).

**Table 8:** Literature review

Study	Inference
Freudenberg S et al [6]	They observed that in situations where superior results of hernia repair depend on the use of a mesh prosthesis but where commercial material is not available or affordable, the use of a nylon mosquito net may be an alternative.
Freudenberg S et al [7]	They concluded from their study that homemade sutures should be recommended to surgeons in countries where the cost of surgical materials often remains an obstacle to life-saving operations.
Conze et al [8]	The researchers observed that the use of the lightweight composite mesh for incisional hernia repair had similar outcomes to polypropylene or polyester mesh, with the exception of a non-significant trend toward increased hernia recurrence.
Klosterhalfen et al [9]	They observed the superiority of the lightweight large porous mesh concept with regard to a reduced number of long-term complications and, particularly, increased comfort and quality of life after hernia repair.
Sørensen & Rosenberg [13]	They concluded that non-commercial meshes for hernioplastic surgery are interesting—especially in a resource-limited setting.
Tongaonkar et al [14]	They emphasised that in economically developing countries like India, the use of cheap mosquito net cloth for the repair of hernias is strongly recommended.
Yang J et al [16]	They concluded that there were no significant differences in outcomes between repairs using low-cost and commercial mesh.
Sanders DL et al [17]	The authors concluded that the in-vitro infection risk of mosquito nets is not significantly different from commonly used monofilament polypropylene commercial prosthetics and is in fact lower than commonly used commercial multifilament mesh.
Sanders DL et al [18]	The authors emphasised that the material and mechanical properties of the polyethylene mosquito net are substantially equivalent to those of commonly used lightweight commercial meshes.
Patterson T et al [19]	They concluded that there was no significant difference between the commercial mesh group and the mosquito net mesh group for pooled (odds ratio 0.93 (0.63, 1.35)) and individual adverse event rates.
Clarke MG et al [21]	They concluded that polyester mosquito net mesh represents a cost-effective alternative to commercial meshes in developing countries with a relatively low rate of early complications and similar short-term recurrence rates.
Kiss A et al [22]	The researchers concluded that the easy use of analysed low-cost material and the demonstrated safety of these sterilised prostheses are two important factors that make them excellent solutions in poor and rural areas such as Southern Sudan.

Limitations

Firstly, the sample size is small. A larger sample size, including more patients, may be useful in the prediction of the results. Secondly, since many patients were lost during follow-up, it would be more meaningful if the study period included a long-term follow-up of the operated patients.

## Conclusions

This study showed that there was not a single instance of mesh rejection when an inguinal hernia was repaired with sterile mosquito net mesh. When compared to traditional polypropylene mesh repair, using the sterile mosquito net mesh resulted in considerable cost savings per operation. In environments with limited resources, using mosquito net mesh for hernioplasty is reasonable, acceptable, doable, and economical.
